# Perspectives of patients with metastatic breast cancer on physical exercise programs: results from a survey in five European countries

**DOI:** 10.1007/s00520-023-08124-4

**Published:** 2023-11-13

**Authors:** Maike G. Sweegers, Johanna Depenbusch, Caroline S. Kampshoff, Neil K. Aaronson, Anouk Hiensch, Yvonne Wengström, Malin Backman, Nadira Gunasekara, Dorothea Clauss, Mireia Pelaez, Milena Lachowicz, Anne M. May, Karen Steindorf, Martijn M. Stuiver, the PERSPECTIVE survey group, Haritz Arrieta, María Gutiérrez Toribio, María López Santillan, Jolien Tol, Wolfram Malter, Julian Puppe

**Affiliations:** 1 Center for Quality of Life, Netherlands Cancer Institute/Antoni van Leeuwenhoek Hospital, Amsterdam, The Netherlands; 2 Department of Psychosocial Research and Epidemiology, Netherlands Cancer Institute/Antoni van Leeuwenhoek Hospital, Amsterdam, The Netherlands; 3 Division of Physical Activity, Prevention and Cancer, German Cancer Research Center (DKFZ) and National Center for Tumor Diseases (NCT), Heidelberg, Germany; 4 Julius Center for Health Sciences and Primary Care, University Medical Center Utrecht, Utrecht University, Utrecht, The Netherlands; 5 Division of Nursing, Department of Neurobiology, Care Sciences, and Society, Karolinska Institute, and Karolinska Comprehensive Cancer Center, Karolinska University Hospital, Stockholm, Sweden; 6 Department for Molecular and Cellular Sports Medicine, German Sport University Cologne, Cologne, Germany; 7 R&D department, Fundación Onkologikoa, Donostia-San Sebastian, Spain and Faculty of Health Sciences, Universidad Europea del Atlántico, Santander, Spain; 8 Department of Oncology and Radiotherapy, Medical University of Gdańsk, Gdańsk, Poland

**Keywords:** Exercise, Patient perspectives, Metastatic breast cancer, Cancer survivorship

## Abstract

**Background:**

To successfully implement exercise programs for patients with metastatic breast cancer (MBC), services and patient education should consider patients’ knowledge, preferences, values, and goals. Hence, gaining insight into their perspectives on exercise and exercise programming is important.

**Method:**

In this cross-sectional survey, we recruited patients with MBC from the Netherlands, Germany, Poland, Spain, and Sweden. We collected data on patients’ knowledge and skills about exercise and outcome expectations. We identified barriers to and facilitators of participation in exercise programs, and patients’ preferences for program content and modes of exercise delivery.

**Results:**

A total of 420 patients participated in the survey. Respondents were, on average, 56.5 years old (SD 10.8) and 70% had bone metastases. Sixty-eight percent reported sufficient skills to engage in aerobic exercise, but only 35% did so for resistance exercise. Respondents expected exercise to have multiple physical benefits, but a few patients expected exercise to worsen their pain (5%). Not having access to an exercise program for cancer patients (27%), feeling too tired (23%), and/or weak (23%) were the most often reported barriers. Facilitators for exercising regularly were previous positive physical (72%) and emotional (68%) experiences with exercising, and receiving personalized advice from a physiotherapist or sport/fitness instructor (62%). Patients were most interested in walking and preferred exercising at a public gym, although there were differences by country. Fifty-seven percent did not know whether their insurance company reimburses exercise programs and only 9% would be willing to pay more than €50 per month to participate.

**Conclusion:**

A large percentage of patients with MBC lack the skills to engage in regular exercise as recommended by exercise guidelines for people with cancer. Patients may benefit from personalized advice and appropriate training facilities to overcome barriers. When implementing exercise interventions, attention should be given to reimbursement and the relatively low willingness-to-pay.

**Supplementary Information:**

The online version contains supplementary material available at 10.1007/s00520-023-08124-4.

## Introduction

Despite improvements in early diagnosis and treatment of breast cancer, approximately 30% of patients initially diagnosed with early stage breast cancer eventually develop metastatic disease [1]. Although current advances in therapy have extended the survival time in patients with MBC [2], these patients still suffer from (long-term) symptoms and side effects related to the disease and its treatment. These include, among others, fatigue, decreased physical functioning, anxiety, and depression [3–5]. All of these symptoms negatively affect patients’ quality of life.

In the curative setting, exercise has positive effects in breast cancer patients in terms of reducing fatigue [6] and improving psychological symptoms such as depression and low self-esteem [7–9]. For metastatic disease, there is limited evidence on the potential effects of exercise. Given the lack of alternatives for reducing fatigue and the pressing unmet need to improve the quality of life of patients with advanced cancer, exercise represents a safe and promising intervention [10].

Patients with cancer who are eligible for an exercise program will weigh, overtly or implicitly, the perceived facilitators of and barriers to taking part in such a program. Facilitators may include the availability of exercise programs, social support, and the belief that exercise will improve health and daily functioning [11–13]. Barriers may include lack of motivation, costs, and travel time [11–13]. To successfully implement exercise programs for patients with MBC, it is important to gain insight into their goals, values, and barriers. This will ensure that education, counseling, and exercise programming meet patients’ needs.

Views about exercise may differ across patients from different European countries. Therefore, the current study aimed to delve into the perspectives on exercise after a diagnosis of MBC among patients from five European countries, and explore inter-country differences. More specifically, we investigated patients’ overall attitude toward exercising following a diagnosis of MBC, their perceived exercise competence, barriers to and facilitators of participation in exercise programs, patients’ goals and expectations regarding the potential benefits of exercise, and their preferences regarding exercise program attributes.

## Method

### Setting

The current study is a subproject of PREFERABLE, a European Commission Research & Innovation Horizon 2020 project (grant agreement No. 825677) aimed at improving the standard of care in patients with MBC. The results of the survey complement the randomized EFFECT trial (NL69600.041.19), which evaluates the efficacy of physical exercise in reducing fatigue and improving health-related quality of life of patients with MBC [14], and the PERSPECTIVE focus group study, an in-depth, qualitative investigation of patients’ perspectives toward exercise [13].

The institutional review boards of all coordinating centers (Germany: Medical faculty of the University of Heidelberg and Deutsche Sporthochschule Köln; the Netherlands: Netherlands Cancer Institute; Poland: Gdanski Uniwersytet Medyczny; Spain: Eusko Jaurlaritza; Sweden: Karolinska Institutet) have reviewed and approved the study.

### Patient recruitment

Patients were eligible if they were diagnosed with histologically confirmed MBC, were aged ≥18 years, had an Eastern Cooperative Oncology Group (ECOG) performance status score of ≤2, and had sufficient command of any of the languages in which the questionnaire was available (German, Dutch, Polish, Spanish, Swedish). Patients with a life expectancy of <6 months, patients who were not able to perform basic activities of daily living, or had cognitive problems that precluded the completion of a questionnaire were excluded from the study. We aimed for inclusion of 100 patients per country, resulting in a target sample size of 500 respondents.

Recruitment started in April 2020 and was completed in March 2022. Potentially eligible patients were identified via hospital registries, and the treating physician or study personnel checked eligibility criteria. Eligible patients were informed about the study either by letter or during a regular follow-up visit with their oncologist or nurse. Patients who expressed interest were asked for permission to be contacted by study personnel who then informed the patients in more detail about the purpose of the survey and shared a unique link to an online portal where participants could complete the survey (Castor EDC [15]). When preferred, patients received a paper version of the survey, which was entered manually in the database by the local researchers. Informed consent was obtained from all patients, either on paper or online.

In August 2021, after 323 patients had completed the questionnaire, we introduced an open recruitment strategy as a supplement to the on-site recruitment strategy. A study flyer was distributed among patient organizations through Europa Donna, the European Breast Cancer Coalition (the Netherlands, Poland, Spain, and Sweden), and mamazone (Germany), who presented the flyer on their website or in a newsletter. The flyer included study information and a request to contact the study team if patients were interested in participating. In December 2021, we shared an open link via Europa Donna, mamazone, and Twitter to direct patients to an introduction page of the survey. For patients responding to this open recruitment strategy, the questionnaire included questions about the participant’s diagnosis and performance status to check for eligibility. When the response to one of these questions did not meet the inclusion criteria, the participant was not able to continue with the questionnaire. We stopped active recruitment on March 31st, 2022. The last respondent completed the questionnaire on May 5th, 2022, after which the survey was taken off-line.

### Data collection

We used a cross-sectional study design in five European countries that together reflect a geographic, cultural, and health systems diversity in Europe: Germany, the Netherlands, Poland, Spain, and Sweden. For validated questionnaires, we used the official translations. Study specific questions were forward translated from English into the target language by each participating site and backward translations were compared to the original wording of the parent questionnaire. The electronic questionnaire was pre-tested by up to three participants per country. Participants completed the survey and a debriefing interview. Issues related to dependencies, response options, and definitions were resolved before the survey was made available to all participants in all countries. The survey included items on demographic and clinical characteristics, physical activity (assessed using the Godin-Shephard Leisure-Time Physical Activity Questionnaire) [16], fatigue (assessed using the EORTC QLQ-FA12 questionnaire) [17], and quality of life (assessed using the EORTC QLQ-C30 questionnaire) [18]. For physical functioning, the domain specific T-score was calculated using software of the EORTC [19, 20]. We specifically instructed respondents to answer the questions about physical activity based on their situation before the coronavirus outbreak.

The study-specific questions in the survey and the response options are presented in Supplementary Table 1. *Patients’ overall attitude* toward exercise was investigated by asking the participants to rate six cognitive and affective aspects on a 7-point Likert scale (−3 to 3) [21]. *Patients’ exercise competence* was evaluated based on their responses to four statements evaluating their exercise knowledge and skills on a 5-point adjective scale ranging from “strongly disagree” to “strongly agree” [22]. In addition, respondents were asked to indicate the minutes of moderate-intensity physical exercise and the number of resistance training sessions per week they believed are needed to gain health benefits [23]. Possible *barriers and facilitators* to engaging in regular exercise were rated from “not at all” to “very much,” on a 5-point adjective scale [24, 25]. Respondents were asked to rank three possible *goals* they would like to achieve by participating in a supervised exercise program. *Patients’ expectations* about exercise effects were evaluated on a 7-point Likert scale, asking participants to rate the likelihood of possible outcomes from −3 “extremely unlikely” to 3 “extremely likely” and the expected effect of exercise on different symptoms from −3 “significantly worsen” to 3 “significantly improve” [26]. In addition, respondents were asked how important the outcomes were to them (unimportant to very important, 0 to 2). Exercise *preferences* were assessed by asking respondents to rank three types of exercise they would be most interested and they were asked whether (yes/no) they preferred light, moderate, or vigorous intensity exercise [27]. Additionally, respondents were asked about their preferred exercise session duration, frequency, location, and supervision, and with whom they would prefer to exercise [27]. For these questions, pre-defined lists of possible responses were available, as well as an “I don’t know/Unsure” and “no preference” option. Finally, respondents were asked whether their current health insurance reimburses exercise programs for patients with cancer and how much they would be willing to pay out of pocket for participation in an exercise program.

### Statistical analysis

Questionnaires with less than 10% completion rate were discarded as these only included responses to demographic questions. For the remaining questionnaires, descriptive statistics characterizing the sample are presented as mean and standard deviation (SD), median and interquartile range (IQR), or frequencies and percentages. For questionnaire outcomes, 95% confidence intervals (95%CI) were calculated. We examined internal consistency using Cronbach’s alpha for the items assessing general attitude (*α* =0.80) and outcome expectation (*α* =0.87) before calculating an overall (mean) score for these constructs. To identify perceived exercise competence, we calculated the percentage of participants responding to the questions with either “agree” or “strongly agree.” To identify the most preferred goal and exercise type, we calculated the percentage of participants responding to each item as either first, second or third preference. Participants’ responses to the questions about outcome expectations and importance of those outcomes were multiplied, and subsequently summed to create a value representing each participant’s overall expected net-benefit of exercise (range: −78 to 78) with higher scores representing more benefit. To identify the main barriers and facilitators, we calculated the frequencies of items that were endorsed with either “quite a bit” or “very much.” We combined response categories for willingness to pay to: max 25 Euros per month, max 50 Euros per month, more than 50 Euros per month, unsure, and not willing to pay anything. Differences between countries were investigated using a Kruskal-Wallis test (ordinal outcomes) or Fisher exact test (nominal outcomes). In case of a significant result (*p*<0.05), pairwise comparisons were performed as post hoc analyses to identify between country differences, in which *p*-values were adjusted according to Benjamini and Hochberg to control for type 1 errors due to multiple comparisons [28]. All analyses were performed using R (version 4.2.1).

## Results

Four-hundred twenty patients participated, of whom 111 (26.4%) were from the Netherlands, 99 (23.6%) from Spain, 98 (23.3%) from Germany, 64 (15.2%) from Poland, and 48 (11.4%) from Sweden. The open-recruitment strategy accounted for 6% of the respondents. Demographic and clinical characteristics are presented in Table 1. In short, the mean age of the respondents was 56.5 (SD 10.8) years, and mean body mass index was 25.6 (SD 5.0). Most of the respondents had bone metastases (70.0%) and some reported liver (39.8%) or lung (27.9%) metastases. Many participants were receiving treatment, most often hormone therapy (56.7%), targeted therapy (40.7%), or chemotherapy (40.5%). Respondents reported a median of 33 min of moderate intensity aerobic physical activity per week (IQR 0–120 min), median global quality of life of 67 (IQR 50–83), and median physical fatigue of 33 (IQR 13–47).

**Table 1:** Descriptives of survey participants (*N* = 420)

	*N, Mdn* or *M*	%, *SD or IQR*
Age^a^	56.5	10.8
Gender, male	4	1
Body mass index^a^	25.6	5.0
Country		
Germany	98	23.3
The Netherlands	111	26.4
Poland	64	15.2
Spain	99	23.6
Sweden	48	11.4
Area of residence		
Urban	233	55.5
Suburban	122	29.0
Rural	64	15.2
Not sure	1	0.2
Marital status		
Married/living with a partner	299	71.2
Divorced/separated	42	10.0
Unmarried/single	43	10.2
Widowed	36	8.6
Highest educational level^c^		
Academic education	177	42.1
Higher education	115	27.4
Middle education	103	24.5
No or basic education	24	5.7
Missing	1	0.2
Current employment status		
Employed	188	44.3
Sick leave	104	24.8
Full-time employee/entrepreneur	25	6.0
Part-time employee/entrepreneur	58	13.8
Missing	1	0.2
Not employed	232	55.7
Location of metastases^d^		
Bone	294	70.0
Liver	167	39.8
Lung	117	27.9
Brain	31	7.4
Other	118	28.1
Missing	3	0.7
Current treatment^d^		
Chemotherapy	170	40.5
Radiotherapy	52	12.4
Hormone therapy	238	56.7
Targeted therapy	171	40.7
Bisphosphonate	132	31.4
Missing	12	2.9
Comorbidities^d^		
Heart disease	25	6.0
Liver disease	33	7.9
High blood pressure	78	18.6
Diabetes mellitus	18	4.3
Back pain	124	29.5
Depression	53	12.6
Missing	4	1.0
Current physical activity (min/week)^b^		
Light intensity aerobic exercise	60	0–140
Moderate intensity aerobic exercise	33	0–120
Vigorous intensity aerobic exercise	0	0–45
Strength/resistance exercise	0	0–12
Quality of life^b,e^		
Global quality of life	67	50–83
Physical functioning T-score^a,g^	45	9
Role functioning	67	50–100
Emotional functioning	67	50–83
Cognitive functioning	83	67–100
Social functioning	67	50–100
Fatigue^b,f^		
Physical fatigue	33	13–47
Emotional fatigue	22	0–42
Cognitive fatigue	0	0–33
Interference with daily life	33	0–33
Social sequalae	0	0–33

^a^Displayed as mean (*M*) and standard deviation (*SD*)

^b^Displayed as median (Mdn) and interquartile range (IQR)

^c^Academic education: bachelor degree or higher (according to Europe-wide Bologna process); higher education: degree qualifying for university; middle education: degree qualifying for further vocational training

^d^Multiple answers possible

^e^Measured using the EORTC QLQ-C30 questionnaire. Scales ranging from 0 to 100, with higher values indicate higher functioning

^f^Measured using the EORTC QLQ-FA12 questionnaire. Scales ranging from 0 to 100, with higher values indicate higher levels of fatigue

^g^Linearly transformed to a 0–100 score, with 50 representing a European general population mean

Results on patients’ perspectives and inter-country differences are detailed in Table 2. Respondents had a positive general attitude toward exercising after their MBC diagnosis (median: 2.3, 95%CI: 2.2;2.3). This differed between countries (*p* = .008), with Swedish participants having a significantly more positive attitude toward exercising compared to Polish participants.

**Table 2:** Survey outcomes

Construct	Item	Value
General attitude	*Overall mean score (-3 to 3)*	2.33, 95% CI = 2.17;2.33
	*Difference between countries* ^*1*^	*P* = .008
	Germany	2.17, 95%CI: 2.00;2.33
	The Netherlands	2.33, 95%CI: 2.00;2.50
	Poland	1.83, 95%CI: 1.50;2.17 (ref)
	Spain	2.33, 95%CI: 2.00;2.50
	Sweden	2.50, 95%CI: 2.33;2.83*
Competence	*Knowledge – amount of exercise necessary to gain health benefits*	0.51, 95%CI: 0.46;0.56
	*Differences between countries* ^*2*^	*P* < .001
	Germany	0.66, 95%CI: 0.56;0.75*
	The Netherlands	0.56, 95%CI: 0.46;0.65*
	Poland	0.37, 95%CI: 0.25;0.50
	Spain	0.32, 95%CI: 0.23;0.43 (ref)
	Sweden	0.64, 95%CI: 0.48;0.77*
	*Knowledge – intensity of exercise necessary to gain health benefits*	0.43, 95%CI: 0.39;0.48
	*Differences between countries* ^*2*^	*P* < .001
	Germany	0.53, 95%CI: 0.42;0.63*
	The Netherlands	0.51, 95%CI: 0.41;0.61*
	Poland	0.36, 95%CI: 0.24;0.49
	Spain	0.27, 95%CI: 0.19;0.37 (ref)
	Sweden	0.51, 95%CI: 0.36;0.66*
	*Competence – skills necessary to engage in aerobic exercise*	0.68, 95%CI: 0.63;0.72
	*Differences between countries* ^*2*^	*P* < .001
	Germany	0.51, 95%CI: 0.40;0.61 (ref)
	The Netherlands	0.76, 95%CI: 0.67;0.84*
	Poland	0.61, 95%CI: 0.47;0.73
	Spain	0.71, 95%CI: 0.61;0.79*
	Sweden	0.85, 95%CI: 0.71;0.93*
	*Competence – skills necessary to engage in resistance exercise*	0.35, 95%CI: 0.31;0.40
	*Differences between countries* ^*2*^	*P* = .01
	Germany	0.40, 95%CI: 0.30;0.50
	The Netherlands	0.33, 95%CI: 0.24;0.43
	Poland	0.26, 95%CI: 0.16;0.39 (ref)
	Spain	0.30, 95%CI: 0.21;0.41
	Sweden	0.55, 95% CI: 0.40;0.70*
	*Knowledge – 140-160 minutes per week of physical exercise needed to achieve health benefits*	0.08, 95%CI: 0.06;0.11
	*Knowledge – 2-3 sessions per week of activities to increase muscle strength needed to achieve health benefits*	0.69, 95%CI: 0.64;0.74
Barriers	*Overall top three barriers*	
	No access to a specialized exercise program for cancer patients	0.27, 95%CI: 0.23; 0.31
	Feeling too weak	0.23, 95%CI: 0.19; 0.28
	Tiredness	0.23, 95%CI: 0.19; 0.28
	*Most endorsed barrier per country* ^*3*^	
	Germany – Feeling too weak	0.28, 95%CI: 0.20;0.39
	The Netherlands – Tiredness	0.26, 95%CI: 0.18;0.35
	Poland – Fear of falls	0.33, 95%CI: 0.22;0.46
	Spain – No access to a specialized exercise program	0.40, 95%CI: 0.31;0.51
	Sweden – Not having an appropriate place to exercise	0.19, 95%CI: 0.10;0.34
Facilitators	*Overall top three facilitators*	
	Having previous positive physical experiences	0.72, 95%CI: 0.68;0.76
	Having previous positive emotional experiences	0.68, 95%CI: 0.63;0.72
	Personalized advice from a physiotherapist or fitness instructor	0.62, 95%CI: 0.57;0.66
	*Most endorsed facilitator per country* ^*3*^	
	Germany – Having previous positive physical experiences	0.82, 95%CI: 0.73;0.89
	The Netherlands – Having previous positive physical experiences	0.64, 95%CI: 0.54;0.73
	Poland – Exercise recommendations from my doctor	0.68, 95%CI: 0.55;0.79
	Spain – Having previous positive physical experiences	0.68, 95%CI: 0.58;0.77
	Sweden – Having previous positive physical experiences	0.89, 95%CI: 0.76;0.96
Goals	*Overall top three goals*	
	Maintain or improve endurance	0.69, 95%CI: 0.64;0.74
	Maintain or improve muscle strength	0.60, 95%CI: 0.55;0.65
	Reduce feelings of fatigue	0.43, 95%CI: 0.38;0.48
	*Most endorsed goal per country*^*3*^	
	Germany – Maintain or improve endurance	0.78, 95%CI: 0.68;0.85
	The Netherlands – Maintain or improve endurance	0.80, 95%CI: 0.71;0.87
	Poland – Maintain or improve endurance	0.65, 95%CI: 0.51;0.76
	Spain – Reduce limitations in daily activities	0.54, 95%CI: 0.43;0.64
	Sweden – Maintain or improve muscle strength	0.83, 95%CI: 0.69;0.92
Expectations	*Overall mean score (-3 to 3)*	1.92, 95%CI: 1.77;2.00
	*Difference between countries*^*1*^	*P* < .001
	Germany	1.92, 95%CI: 1.62;2.15*
	The Netherlands	1.54, 95%CI: 1.31;1.77 (ref)
	Poland	1.92, 95%CI: 1.69;2.31*
	Spain	2.08, 95%CI: 1.85;2.38*
	Sweden	2.08, 95%CI: 2.00;2.38*
Preferred exercise type	*Overall top three exercise types*	
	Walking	0.65, 95%CI: 0.60;0.69
	Mind-body exercises	0.47, 95%CI: 0.42;0.52
	Flexibility exercises	0.44, 95%CI: 0.39;0.49
	*Most preferred exercise type per country*^*3*^	
	Germany – Flexibility exercises	0.59, 95%CI: 0.48;0.69
	The Netherlands – Walking	0.71, 95%CI: 0.61;0.79
	Poland – Walking	0.74, 95%CI: 0.61;0.84
	Spain – Walking	0.87, 95%CI: 0.79;0.93
	Sweden – Walking	0.62, 95%CI: 0.46;0.75
Preferred exercise intensity	*Exercise intensity (multiple answers possible)*	
	Light intensity exercise	0.25, 95%CI: 0.21;0.30
	Moderate intensity exercise	0.59, 95%CI: 0.54; 0.64
	Vigorous intensity exercise	0.15, 95%CI: 0.12; 0.19
	No preference	0.04, 95%CI: 0.02; 0.06
	Don’t know	0.07, 95%CI: 0.04; 0.10
Preferred exercise session duration	*Overall most preferred exercise session duration*	
	Over 45 minutes	0.26, 95%CI: 0.22;0.30
	*Differences between countries* ^*2*^	*P* = .02
	*Most preferred exercise session duration per country*	
	Germany – Over 45 minutes	0.29, 95%CI: 0.21;0.39
	The Netherlands – 30-45 minutes	0.24, 95%CI: 0.16;0.33
	Poland – 20-30 minutes	0.24, 95%CI: 0.14;0.36
	Spain – Over 45 minutes	0.30, 95%CI: 0.21;0.41
	Sweden – 30-45 minutes	0.36, 95%CI: 0.23;0.52
Preferred exercise frequency	*Most preferred exercise frequency*	
	Twice a week	0.40, 95%CI: 0.35;0.45
	*Differences between countries* ^*2*^	*P* < .001
	*Most preferred exercise frequency per country*	
	Germany – Twice a week	0.41, 95%CI: 0.31;0.52
	The Netherlands – Twice a week	0.46, 95%CI: 0.37;0.56
	Poland – Twice a week	0.32, 95%CI: 0.21;0.45
	Spain – Twice a week	0.35, 95%CI: 0.26;0.46
	Sweden – Twice a week	0.40, 95%CI: 0.27;0.56
Preferred exercise location	*Most preferred exercise location*	
	Public gym or community sports facility	0.26, 95%CI: 0.21;0.30
	*Differences between countries* ^*2*^	*P* < .001
	*Most preferred exercise location per country*	
	Germany – Public gym or community sports facility	0.34, 95%CI: 0.25;0.45
	The Netherlands – Physiotherapy practice	0.26, 95%CI: 0.18;0.36
	Poland – At home	0.21, 95%CI: 0.11;0.34
	Spain – Outdoors	0.25, 95%CI: 0.17;0.35
	Sweden – Public gym or community sports facility	0.36, 95%CI: 0.23;0.52
Preferred exercise supervision	*Most preferred exercise supervision*	
	Physiotherapist	0.38, 95%CI: 0.33;0.43
	*Differences between countries* ^*2*^	*P* < .001
	*Most preferred exercise supervision per country*	
	Germany – Fitness instructor or exercise professional	0.42, 95%CI: 0.32;0.52
	The Netherlands – Physiotherapist	0.42, 95%CI: 0.33;0.52
	Poland – Physiotherapist	0.52, 95%CI: 0.40;0.65
	Spain – Physiotherapist	0.29, 95%CI: 0.21;0.39
	Sweden – Fitness instructor or exercise professional	0.40, 95%CI: 0.27;0.56
Preference with whom they wished to exercise	*Most preferred exercise company*	
	No preference	0.21, 95%CI: 0.17;0.25
	*Differences between countries* ^*2*^	Not significant
Reimbursement	*Reimbursement*	
	I don’t know	0.57, 95%CI:0.52;0.62
	*Differences between countries* ^*2*^	*P* < .001
	*Reimbursement per country*	
	Germany – I don’t know	0.58, 95%CI: 0.48;0.68
	The Netherlands – I don’t know	0.63, 95%CI: 0.53;0.72
	Poland – I don’t know	0.41, 95%CI: 0.29;0.54
	Spain – I don’t know	0.54, 95%CI: 0.43;0.64
	Sweden – I don’t know	0.69, 95%CI: 0.54;0.81
Costs	*Willingness-to-pay*	
	Max 25 Euros per month	0.36, 95%CI: 0.32;0.41
	*Differences between countries* ^*2*^	*P* < .001
	*Willingness-to-pay per country*	
	Germany – Max 25 Euros per month	0.39, 95%CI: 0.30;0.50
	The Netherlands – Max 25 Euros per month	0.41, 95%CI: 0.32;0.51
	Poland – Max 25 Euros per month	0.42, 95%CI: 0.30;0.55
	Spain – Unsure	0.38, 95%CI: 0.28;0.48
	Sweden – Max 25 Euros per month	0.43, 95%CI: 0.29;0.58

Table presents proportion of participants for the most frequently endorsed response option. (*) significantly different from reference category (ref), with p-values adjusted according to Benjamini & Hochberg

^1^Investigated using Kruskal-Wallis test

^2^Investigated using Fisher exact test

^3^Investigating significant differences between countries was not possible due to the structure of the survey question. Therefore, we provided the overall top 3 responses and the most endorsed response per country

Approximately half of the respondents reported having a good idea of how much exercise they should do to gain health benefits (51%, 95%CI: 46;56), and with what intensity they should exercise (43%, 95%CI: 39;48). *Actual* knowledge about the amount of aerobic exercise needed to gain health benefits was much less, with only 8% (95%CI: 6;11) responding correctly that this should be between 140 and 160 min per week (54% responded <140 min/week, 38% responded >160 min/week). For resistance exercise, 69% (95%CI: 64;74) correctly responded that this should be performed in 2 to 3 sessions per week. Sufficient skills to engage in aerobic exercise were reported by 68% (95%CI: 63;72), but only 35% (95%CI: 31;40) felt they had sufficient skills for performing resistance exercise, with inter-country differences (*p-*values ≤ .01).

**Fig. 1:**
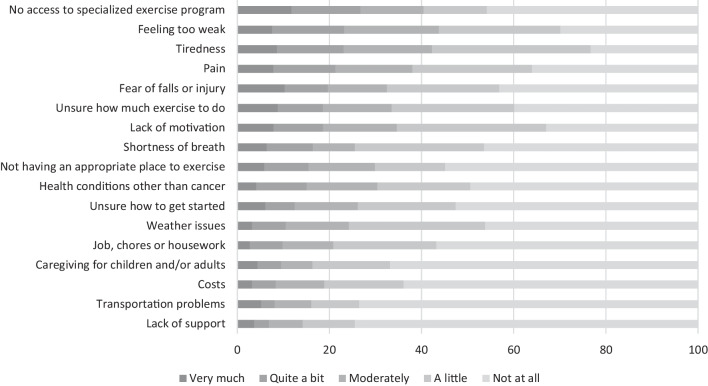
Participant responses (in percentages) regarding barriers standing in the way for exercising on a regular basis

**Fig. 2:**
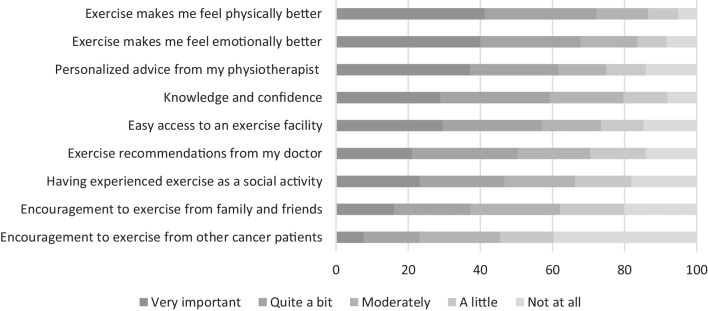
Participant responses (in percentages) regarding reasons to start or continue exercising on a regular basis

Overall, lack of access to specialized exercise programs (27%, 95%CI: 23;31), feeling too weak (23%, 95%CI: 19;28), and tiredness (23%, 95%CI: 19;28) were the main perceived *barriers* to exercising (Fig. 1), with inter-country differences in the primary barrier. The main reasons to start or continue exercising on a regular basis were having previous positive physical (72%, 95%CI: 68;76) or emotional (68%, 95%CI: 63;72) experiences from exercise or receiving personalized advice from a physical therapist (62%, 95%CI: 57;66; Fig. 2). Participants from Poland reported receiving exercise recommendations from their doctor to be the main facilitator. The most frequently reported *goals* were to maintain or improve endurance (69%, 95%CI: 64;74), to maintain or improve muscle strength (60%, 95%CI: 55; 65), and to reduce feelings of fatigue (43%, 95%CI: 38;48), with differences in the main goals reported by participants from different countries.

Respondents had an overall positive outcome expectation toward exercising (median: 1.9, 95%CI: 1.8;2.0), with significant differences between countries (*p* < .001). Nevertheless, participants expressed negative outcome expectations on individual statements, most often for “participating in exercise will help stay at or return to work” (22%, 95%CI: 18;26), “exercise will improve my pain” (5%, 95%CI: 3;8), and “exercise will be enjoyable” (5%, 95%CI: 3;8). Participant’s median overall expected net-benefit of exercise was 41 (95%CI: 37;44).

The most frequently preferred exercise types were walking (65%, 95%CI: 60; 69), mind-body exercise (e.g., yoga, Tai Chi, Pilates; 47%, 95%CI: 42;52), and flexibility exercises (44%, 95%CI: 39;49). For each country separately, the most preferred exercise type was walking, except for Germany where participants preferred flexibility exercises. Most participants endorsed moderate intensity exercise (59%, 95%CI: 54;64). Although the distribution of preferences for exercise frequency varied significantly across countries (*p* < .001), the mostly preferred exercising frequency was the same for all countries, twice per week (40%, 95%CI: 35;45). Most participants preferred to exercise for more than 45 min per session (26%, 95%CI: 22;30), with inter-country differences (*p* = .02). Overall, the most preferred exercise location was at a public gym or community sports facility (26%; 95%CI: 21;30), but Dutch participants most often preferred exercising at a physiotherapy practice, Polish participants at home, and Spanish participants outdoors.

Participants preferred the exercise program to be supervised by a physiotherapist (38%, 95%CI: 33;43), while those from Germany and Sweden most often preferred an exercise program to be supervised by a fitness instructor or exercise professional. Approximately one-fifth of the respondents had no preference regarding with whom they would like to exercise (21%, 95%CI: 17;25). Over half of the participants (57%, 95%CI: 52;62) did not know whether their insurance company reimburses exercise programs. Acceptable out of pocket expenses were maximally 25 Euros per month for 36% (95%CI: 32;41), and only 9% (95%CI: 6;12) were willing to spend more than 50 Euros per month on exercise programs. Full details of preferences per country are available in Supplementary Table 2.

## Discussion

In this multinational survey, patients with MBC generally reported a positive attitude toward exercise. However, their skills were insufficient, and the majority erroneously believed that they knew how much exercise was needed to improve their health [29]. Most frequently reported barriers to exercising regularly were treatment-related side effects such as tiredness, pain, and feeling too weak. Overall, participants expected positive results of exercising for the outcomes they valued, although a small percentage expressed concerns that some of their symptoms might be aggravated. We found inter-country differences regarding attitudes toward exercising and preferences for exercise program attributes. Relatively few respondents reported being aware of whether their health insurance reimburses for the costs of participating in an exercise program.

The attitude toward exercise from patients with MBC does not appear to differ much from that of patients treated with curative intent [30–33]. Although patients with breast cancer generally believe that exercise has positive effects, previously reported barriers to engage in exercise included fear for negative side effects from exercise, cancer- or treatment-related physical complaints, and lack of appropriate facilities, motivation, or skills [34, 35]. Many of these barriers were also reported by patients with MBC in focus group interviews [13, 27] and by participants of the current survey. Patients with advanced disease may need more specific instructions for safe execution of exercises [13], and prefer personalized advice and instructions for exercises that accommodate a changing physical capacity [36]. Increasing evidence suggests that exercise is safe and beneficial for patients with advanced disease [37–39], but high-quality research for MBC patients is still very limited. Our findings highlight the need for better patient education regarding appropriate activity levels as well as individualized exercise programs.

Participants in our study mostly endorsed improving or maintaining endurance and muscle strength. Similar priorities were also observed in a previous Dutch survey study [27]. These outcomes may have intrinsic value to them, or patients with MBC may typically associate exercise with fitness outcomes, which indirectly support the achievement of goals such as symptom control or activities of daily life [30]. In general, respondents to the current survey did not expect exercise to help them stay at or return to work, while evidence of moderate quality suggests positive effects of physical activity interventions on return to work rates in cancer survivors [40]. Whether these results can also be expected in patients with MBC is unclear.

Participants had no clear preference regarding with whom they wished to exercise, which may reflect indifference, but more likely ambivalence. Previous studies have reported that group exercises provide the opportunity to interact with other cancer patients and to talk with patients with similar issues [30, 32]. During focus group interviews with patients with MBC, some patients indicated a preference for interacting with other patients, while others did not want to dwell on their cancer experience and preferred exercising in settings with people from the general population [13]. Overall, preferences reported by our sample regarding exercise frequency, intensity, and duration correspond to the recommended amount of exercise to gain health benefits [29].

Half of the participants were unaware of whether their current health insurer reimburses exercise programs for patients with cancer, which indicates that there is a need for transparent and clear communication about reimbursement for such programs. Although costs were reported to be a barrier by only a small percentage of respondents (8%), the costs of a typical supervised exercise program will likely exceed the willingness to pay for such programs for many participants. More evidence of the positive effects and cost-effectiveness of supervised exercise for patients with MBC may increase the willingness of health insurers to reimburse the costs of these programs in European countries where exercise is currently not (fully) reimbursed.

The inter-country differences we observed suggest that implementation strategies will benefit from a country-specific, tailored approach. For example, in Sweden, implementing face-to-face supervised exercise programs may not be possible given that the limited availability of appropriate places to exercise is a main perceived barrier. Online supervised programs that patients can follow from home might circumvent this issue, but their effectiveness should be further explored [13]. Such an approach might be less suitable for Poland where (initial) face-to-face supervision by an exercise specialist may be required to overcome fear of falls, even though Polish participants considered home to be their preferred exercise location. In addition, in Poland, it is more important than in the other countries to acknowledge the role of the treating physician in providing exercise recommendations, and to shape clinical pathways accordingly. Healthcare professionals play an important role in exercise counseling (assess, advise, refer) and they should advise patients to reach the desired level of exercise to achieve optimal benefits. In addition, additional education may be necessary for physiotherapists or sport/fitness instructors supervising patients with advanced disease [41]. More detailed recommendations for implementation are presented in a living document, available as a public deliverable of the EU Horizon 2020 project via the EU CORDIS website 10.3030/825677.

### Strengths and limitations

To our knowledge, this is the first quantitative study investigating perspectives of patients with MBC on exercise across different European countries. There are some limitations in our study that should be noted. Some of the participating countries have a large surface area with intra-country differences in, among other things, weather conditions and cultural values. We recruited primarily patients living near the participating hospitals. For example, 96% of the Spanish participants were recruited via hospitals located in the Basque region, which is well known for its hiking trails and milder climate compared to other regions of Spain. Because of a lower than expected recruitment rate, we also recruited patients via patient organizations and Twitter. Both recruitment strategies might have introduced a selective response of more exercise-minded patients. Patients were recruited during the corona pandemic, which may have influenced their attitude toward exercise and exercise preferences. From focus group discussions with patients with MBC during the corona pandemic, we know that some patients expressed worries about exercising in enclosed spaces due to possible increased risks for infection [13]. Finally, despite extra efforts and an open-recruitment strategy, we included less participants than our initial target (i.e., 500 participants). Although this may have affected to some extent the statistical power of our cross-country analyses, we have no reason to believe that a larger sample would have significantly affected the outcomes or conclusions of the study.

### Conclusion

The results of the current survey indicate that a large proportion of patients with MBC lacks the necessary skills to engage in regular exercise. Patients may benefit from personalized advice and exercise programs that accommodate a patient’s changing physical capacity. Our findings highlight the need for educational materials on expected benefits of exercising, and help patients to overcome barriers to exercising. When implementing exercise programs for patients with MBC, specific attention should be given to the costs of and reimbursement of exercise programs and implementation strategies may benefit from a country-specific approach.

### Supplementary information

ESM 1 (DOCX 17 kb)

ESM 2 (DOCX 16 kb)

## Data Availability

The authors declare that they have full control of all primary data and that they agree to allow the journal to review the data.

## References

[1] O’Shaughnessy J (2005). Extending survival with chemotherapy in metastatic breast cancer. Oncologist..

[2] Jemal A, Ward EM, Johnson CJ, Cronin KA, Ma J, Ryerson B et al (2017) Annual report to the nation on the status of cancer, 1975-2014, featuring survival. J Natl Cancer Inst 109(9). 10.1093/jnci/djx030 10.1093/jnci/djx030 PMC5409140 28376154

[3] Jones JM, Olson K, Catton P, Catton CN, Fleshner NE, Krzyzanowska MK (2016). Cancer-related fatigue and associated disability in post-treatment cancer survivors. J Cancer Surviv.

[4] Bower JE (2006). Management of cancer-related fatigue. Clin Adv Hematol Oncol.

[5] Reich M, Lesur A, Perdrizet-Chevallier C (2008). Depression, quality of life and breast cancer: a review of the literature. Breast Cancer Res Treat.

[6] Van Vulpen JK, Sweegers MG, Peeters PHM, Courneya KS, Newton RU, Aaronson NK (2020). Moderators of exercise effects on cancer-related fatigue: a meta-analysis of individual patient data. Med Sci Sports Exerc.

[7] Mehnert A, Veers S, Howaldt D, Braumann KM, Koch U, Schulz KH (2011). Effects of a physical exercise rehabilitation group program on anxiety, depression, body image, and health-related quality of life among breast cancer patients. Onkologie..

[8] Ergun M, Eyigor S, Karaca B, Kisim A, Uslu R (2013). Effects of exercise on angiogenesis and apoptosis-related molecules, quality of life, fatigue and depression in breast cancer patients. Eur J Cancer Care (Engl).

[9] Courneya KS, Segal RJ, Mackey JR, Gelmon K, Reid RD, Friedenreich CM (2007). Effects of aerobic and resistance exercise in breast cancer patients receiving adjuvant chemotherapy: a multicenter randomized controlled trial. J Clin Oncol.

[10] Ligibel JA, Bohlke K, Alfano CM (2022). Exercise, diet, and weight management during cancer treatment: ASCO guideline summary and Q&A. JCO Oncol Pract.

[11] Smith-Turchyn J, Allen L, Dart J, Lavigne D, Rooprai S, Dempster H (2021). Characterizing the exercise behaviour, preferences, barriers, and facilitators of cancer survivors in a rural Canadian community: a cross-sectional survey. Curr Oncol.

[12] Weller S, Oliffe JL, Campbell KL (2019). Factors associated with exercise preferences, barriers and facilitators of prostate cancer survivors. Eur J Cancer Care (Engl).

[13] Depenbusch J, Sweegers MG, Aaronson NK, Wengstrom Y, Backman M, Arraras JI (2023). PERSPECTIVEs on supervised exercise programs in people with metastatic breast cancer- a qualitative study in four European countries. Support Care Cancer.

[14] Hiensch AE, Monninkhof EM, Schmidt ME, Zopf EM, Bolam KA, Aaronson NK (2022). Design of a multinational randomized controlled trial to assess the effects of structured and individualized exercise in patients with metastatic breast cancer on fatigue and quality of life: the EFFECT study. Trials..

[15] Castor EDC (2019) Castor Electronic Data Capture. [online] Available at: https://castoredc.com

[16] Amireault S, Godin G, Lacombe J, Sabiston CM (2015). Validation of the Godin-Shephard Leisure-Time Physical Activity Questionnaire classification coding system using accelerometer assessment among breast cancer survivors. J Cancer Surviv.

[17] Weis J, Tomaszewski KA, Hammerlid E, Ignacio Arraras J, Conroy T, Lanceley A et al (2017) International psychometric validation of an EORTC quality of life module measuring cancer related fatigue (EORTC QLQ-FA12). J Natl Cancer Inst 109(5). 10.1093/jnci/djw273 10.1093/jnci/djw273 28376231

[18] Aaronson NK, Ahmedzai S, Bergman B, Bullinger M, Cull A, Duez NJ (1993). The European Organization for Research and Treatment of Cancer QLQ-C30: a quality-of-life instrument for use in international clinical trials in oncology. J Natl Cancer Inst.

[19] Liegl G, Petersen MA, Groenvold M, Aaronson NK, Costantini A, Fayers PM (2019). Establishing the European Norm for the health-related quality of life domains of the computer-adaptive test EORTC CAT Core. Eur J Cancer.

[20] Petersen MA, Groenvold M, Aaronson NK, Chie WC, Conroy T, Costantini A (2011). Development of computerized adaptive testing (CAT) for the EORTC QLQ-C30 physical functioning dimension. Qual Life Res.

[21] Speed-Andrews AE, McGowan EL, Rhodes RE, Blanchard CM, Culos-Reed SN, Friedenreich CM (2014). Identification and evaluation of the salient physical activity beliefs of colorectal cancer survivors. Cancer Nurs.

[22] Short CE, James EL, Girgis A, McElduff P, Plotnikoff RC (2012). Move more for life: the protocol for a randomised efficacy trial of a tailored-print physical activity intervention for post-treatment breast cancer survivors. BMC Cancer.

[23] Florindo AA, Brownson RC, Mielke GI, Gomes GA, Parra DC, Siqueira FV (2015). Association of knowledge, preventive counseling and personal health behaviors on physical activity and consumption of fruits or vegetables in community health workers. BMC Public Health.

[24] Cadmus-Bertram LA, Gorzelitz JS, Dorn DC, Malecki KMC (2020). Understanding the physical activity needs and interests of inactive and active rural women: a cross-sectional study of barriers, opportunities, and intervention preferences. J Behav Med.

[25] Gustaw T, Schoo E, Barbalinardo C, Rodrigues N, Zameni Y, Motta VN et al (2017) Physical activity in solid organ transplant recipients: participation, predictors, barriers, and facilitators. Clin Transpl 31(4). 10.1111/ctr.12929 10.1111/ctr.12929 28185297

[26] Plotnikoff RC, Blanchard CM, Hotz SB, Rhodes R (2001) Validation of the decisional balance scales in the exercise domain from the transtheoretical model: a longitudinal test. Meas Phys Educ Exerc Sci 5(191)

[27] Ten Tusscher MR, Groen WG, Geleijn E, Sonke GS, Konings IR, Van der Vorst MJ (2019). Physical problems, functional limitations, and preferences for physical therapist-guided exercise programs among Dutch patients with metastatic breast cancer: a mixed methods study. Support Care Cancer.

[28] Benjamini Y, Hochberg Y (1995). Controlling the false discovery rate: a practical and powerful approach to multiple testing. J R Stat Soc.

[29] Campbell KL, Winters-Stone KM, Wiskemann J, May AM, Schwartz AL, Courneya KS (2019). Exercise guidelines for cancer survivors: consensus statement from international multidisciplinary roundtable. Med Sci Sports Exerc.

[30] Browall M, Mijwel S, Rundqvist H, Wengstrom Y (2018). Physical activity during and after adjuvant treatment for breast cancer: an integrative review of women’s experiences. Integr Cancer Ther.

[31] Husebo AM, Allan H, Karlsen B, Soreide JA, Bru E (2015). Exercise: a path to wellness during adjuvant chemotherapy for breast cancer?. Cancer Nurs.

[32] Bulmer SM, Howell J, Ackerman L, Fedric R (2012). Women’s perceived benefits of exercise during and after breast cancer treatment. Women Health.

[33] Whitehead S, Lavelle K (2009). Older breast cancer survivors’ views and preferences for physical activity. Qual Health Res.

[34] Sander AP, Wilson J, Izzo N, Mountford SA, Hayes KW (2012). Factors that affect decisions about physical activity and exercise in survivors of breast cancer: a qualitative study. Phys Ther.

[35] Brunet J, Taran S, Burke S, Sabiston CM (2013). A qualitative exploration of barriers and motivators to physical activity participation in women treated for breast cancer. Disabil Rehabil.

[36] Shallwani SM, Thomas R, King J, Toupin-April K, Poitras S (2023) Perspectives and experiences of leisure-time physical activity in adults with stage 4 cancer: a qualitative interpretive-description study. Disabil Rehabil:1–12. 10.1080/09638288.2023.2200037 10.1080/09638288.2023.2200037 37067063

[37] Nadler MB, Desnoyers A, Langelier DM, Amir E (2019). The effect of exercise on quality of life, fatigue, physical function, and safety in advanced solid tumor cancers: a meta-analysis of randomized control trials. J Pain Symptom Manag.

[38] Chen YJ, Li XX, Ma HK, Zhang X, Wang BW, Guo TT (2020). Exercise training for improving patient-reported outcomes in patients with advanced-stage cancer: a systematic review and meta-analysis. J Pain Symptom Manag.

[39] Rodriguez-Canamero S, Cobo-Cuenca AI, Carmona-Torres JM, Pozuelo-Carrascosa DP, Santacruz-Salas E, Rabanales-Sotos JA (2022). Impact of physical exercise in advanced-stage cancer patients: systematic review and meta-analysis. Cancer Med.

[40] Wilson TN, Nambiema A, Porro B, Descatha A, Aublet-Cuvelier A, Evanoff B et al (2022) Effectiveness of physical activity interventions on return to work after a cancer diagnosis: a systematic review and meta-analysis. J Occup Rehabil. 10.1007/s10926-022-10052-9 10.1007/s10926-022-10052-9 PMC10025244 35779184

[41] Sheill G, Guinan E, L ON, Hevey D, Hussey J. (2018). Physical activity and advanced cancer: the views of chartered physiotherapists in Ireland. Physiother. Theory Pract.

