# Supplementary material for: Perspectives of patients with metastatic breast cancer on physical exercise programs: results from a survey in five European countries
Source: Support Care Cancer. 2023 Nov 13;31(12):694. doi: 10.1007/s00520-023-08124-4 (PMC10643348; doi:10.1007/s00520-023-08124-4)
Supplement: Supplementary file 1 — (DOCX 17 kb) [file 520_2023_8124_MOESM1_ESM.docx]

| Supplementary table 1. Survey questions, response options and values | | | |
| --- | --- | --- | --- |
| Outcome | **Items** | **Response type** | **Values** |
| General attitude | *Please choose the response category that best applies to you*.  Useless-useful,  Harmful-beneficial,  Unpleasant-enjoyable,  Boring-fun,  Difficult-easy,  Unwise-sensible. | 7-point likert scale | -3  -2  -1  0  1  2  3 |
| Competence | *I have a good idea of how much exercise I should do to gain health benefits,*  *I have a good idea of how intensively I should exercise to gain health benefits,*  *I have the skills necessary to engage in aerobic exercise,*  *I have the skills necessary to engage in resistance-based exercise.* | 5-point likert scale | Strongly disagree,  Disagree,  Neutral,  Agree,  Strongly agree. |
|  | *How many minutes per week of moderate-intensity physical exercise do you think are needed to achieve health benefits?*  *How many sessions per week of activities to increase muscle strength do you think are needed to achieve health benefits?* | Numeric | Minutes  Sessions |
| Barriers | *To what extent do the following reasons stand in your way for exercising on a regular basis?*  Feeling too weak,  Health conditions other than cancer,  No access to a specialized exercise program for cancer patients,  Not having an appropriate place to exercise,  Transportation problems,  Difficulty finding time due to caregiving,  Difficulty finding time due to a job, chores or housework,  Weather issues,  Lack of motivation,  Lack of support from friends and family,  Being unsure how to get started,  Being unsure how much exercise they should do,  Costs,  Fear of falls or injury,  Tiredness,  Pain,  Shortness of breath. | 5-point likert scale | Not at all,  A little,  Moderately,  Quite a bit,  Very much. |
| Facilitators | *How important are the following reasons for you to start or continue exercising on a regular basis?*  Having positive physical experiences from exercise in the past,  Having positive emotional experiences from exercise in the past,  Having experienced exercise as a social activity,  Encouragement from friends and family,  Encouragement from other cancer patients,  Recommendations from their doctor,  Knowledge and confidence about exercise,  Easy access to exercise facilities,  Personalized advice from a physiotherapist or fitness instructor. | 5-point likert scale | Not at all,  A little,  Moderately,  Quite a bit,  Very much. |
| Goals | *Which goal(s) would you like to achieve by a supervised exercise program?*  Maintain or improve endurance,  Maintain or improve muscle strength,  Reduce limitations in daily activities,  Reduce feelings of fatigue,  Reduce pain in joints and muscles,  Reduce shortness of breath,  Reduce tension and stress level,  To feel more confident about their body,  No specific goal,  Other. | Rating | First preference,  Second preference,  Third preference. |
| Expectations | *Participating in regular exercise will:*  Improve my ability to perform daily activities,  Be beneficial for the health of my heart and/or lungs,  Help me control their weight,  Increase my muscle strength,  Help me stay at work or return after sick leave,  Be enjoyable,  Increase my self-esteem. | 7-point likert scale | Extremely unlikely,  Very unlikely,  Unlikely,  Neutral,  Likely,  Very likely,  Extremely likely. |
|  | *By participating in regular exercise, I expect that:*  My mood will ..,  My sleep will ..,  My tension or stress will ..,  My fatigue will ..,  My pain will ..,  My lymphedema will … | 7-point likert scale | Significantly worsen,  Very likely worsen,  Likely worsen,  Neutral,  Likely improve,  Very likely improve,  Significantly improve. |
|  | *For me, xxx is….* | 3-point likert scale | Unimportant,  Important,  Very important. |
| Preferred exercise type | *What type of exercise program would you be most interested in?*  Walking,  Cycling,  Strengthening  Exercises,  Flexibility exercises,  Aerobic exercises,  Swimming,  Circuit training,  Mind-body exercises,  Bootcamp activities,  Other sports or sport games,  No preference. | Rating | First preference,  Second preference,  Third preference. |
| Preferred exercise intensity | *What would be your preferred intensity of an exercise program?*  Light intensity exercise,  Moderate intensity exercise,  Vigorous intensity exercise,  No preference,  Don’t know. | checkbox | Yes,  No. |
| Preferred exercise session duration | *How long do you think you would currently be able to participate in an exercise session?* | Radio buttons | Less than 10 minutes,  10-20 minutes,  20-30 minutes,  30-45 minutes,  over 45 minutes  Not sure. |
| Preferred exercise frequency | *How often would you prefer to attend an exercise program?* | Radio buttons | Once every now and then,  Once every two weeks,  Once a week,  Twice a week,  Three times a week, More than three times a week,  No preference,  Not sure. |
| Preferred exercise location | *Where would you prefer an exercise program to take place?* | Radio buttons | At home, Physiotherapy practice,  Cancer center, Hospital,  Public gym or community sports facility,  Outdoors,  No preference,  Not sure. |
| Preferred exercise supervision | *Who would you prefer your exercise program to be supervised by?* | Radio buttons | No one,  Specialist nurse, Physiotherapist, Other healthcare professional,  Fitness instructor or exercise professional,  No preference,  Not sure. |
| Preferred exercise company | *With whom would you prefer to exercise?* | Radio buttons | No one,  Cancer patients, General public, Family,  Friends,  No preference,  Not sure. |
| Reimbursement | *Does your current insurance company reimburse exercise or rehabilitation programs for people with cancer?* | Radio buttons | Yes, but under specific conditions,  No, my insurance does not cover exercise or rehabilitation programs for people with cancer,  I don’t know. |
| Costs | *How much would you be willing to pay for participation in an exercise program?* | Radio buttons | I am not willing to pay anything at all,  Max 5 Euros per month,  Max 15 Euros per month,  Max 25 Euros per month,  Max 50 Euros per month,  Max 75 Euros per month,  Max 100 Euros per month,  Max 150 Euros per month,  Max 200 Euros per month,  Max 250 Euros per month,  Not sure. |
