# Supplementary material for: Perspectives of patients with metastatic breast cancer on physical exercise programs: results from a survey in five European countries
Source: Support Care Cancer. 2023 Nov 13;31(12):694. doi: 10.1007/s00520-023-08124-4 (PMC10643348; doi:10.1007/s00520-023-08124-4)
Supplement: Supplementary file 2 — (DOCX 16 kb) [file 520_2023_8124_MOESM2_ESM.docx]

| Supplementary Table 2. Survey outcomes per country | | |
| --- | --- | --- |
| Preferred exercise type | *Top three exercise types*  *Germany*  Flexibility exercises  Mindbody exercises  Cycling/ Swimming  *The Netherlands*  Walking  Cycling  Strength exercises  *Poland*  Walking  Cycling  Strength exercises  *Spain*  Walking  Mindbody exercises  Flexibility exercises  *Sweden*  Walking  Strength exercises  Mindbody exercises | 0.59, 95%CI: 0.48;0.69  0.51, 95%CI: 0.40;0.61  0.40, 95%CI: 0.30;0.51  0.71, 95%CI: 0.61;0.79  0.53, 95%CI: 0.43;0.63  0.45, 95%CI: 0.35;0.55  0.74, 95%CI: 0.61;0.84  0.55, 95%CI: 0.42;0.67  0.53, 95%CI: 0.40;0.66  0.87, 95%CI: 0.79;0.93  0.63, 95%CI: 0.53;0.73  0.61, 95%CI: 0.50;0.71  0.62, 95%CI: 0.46;0.75  0.53, 95%CI: 0.38;0.68  0.45, 95%CI: 0.30;0.60 |
| Preferred exercise intensity | *Exercise intensity (multiple answers possible)*  *Germany*  Light intensity exercise  Moderate intensity exercise  Vigorous intensity exercise  No preference  Don’t know  *The Netherlands*  Light intensity exercise  Moderate intensity exercise  Vigorous intensity exercise  No preference  Don’t know  *Poland*  Light intensity exercise  Moderate intensity exercise  Vigorous intensity exercise  No preference  Don’t know  *Spain*  Light intensity exercise  Moderate intensity exercise  Vigorous intensity exercise  No preference  Don’t know  *Sweden*  Light intensity exercise  Moderate intensity exercise  Vigorous intensity exercise  No preference  Don’t know | 0.29, 95%CI: 0.20;0.39  0.58, 95%CI: 0.47;0.68  0.20, 95%CI: 0.12;0.29  0.02, 95%CI: 0.004;0.08  0.08, 95%CI: 0.04;0.16  0.15, 95%CI: 0.09;0.23  0.62, 95%CI: 0.52;0.71  0.13, 95%CI: 0.08;0.21  0.06, 95%CI: 0.02;0.12  0.10, 95%CI: 0.05;0.18  0.38, 95%CI: 0.26;0.51  0.48, 95%CI: 0.35;0.60  0.11, 95%CI: 0.05;0.22  0.05, 95%CI: 0.01;0.14  0.02, 95%CI: 0.001;0.10  0.36, 95%CI: 0.27;0.47  0.59, 95%CI: 0.48;0.69  0.05, 95%CI: 0.02;0.12  0.03, 95%CI: 0.01;0.09  0.07, 95%CI: 0.03;0.15  0.02, 95%CI: 0.001;0.13  0.74, 95%CI: 0.59;0.86  0.34, 95%CI: 0.21;0.49  0.02, 95%CI: 0.001;0.13  0.00, 95%CI: 0.00;0.09 |
| Preferred exercise session duration | *Top three exercise session duration*  *Germany*  Over 45 minutes  20-30 minutes  30-45 minutes  *The Netherlands*  30-45 minutes  10-20 minutes  Over 45 minutes/20-30 minutes  *Poland*  20-30 minutes  30-45 minutes  Over 45 minutes  *Spain*  Over 45 minutes  30-45 minutes  20-30 minutes  *Sweden*  30-45 minutes  Over 45 minutes  20-30 minutes | 0.29, 95%CI: 0.21;0.39  0.21, 95%CI: 0.14;0.32  0.19, 95%CI: 0.12;0.28  0.24, 95%CI: 0.16;0.33  0.23, 95%CI: 0.15;0.32  0.19, 95%CI: 0.12;0.28  0.24, 95%CI: 0.14;0.36  0.22, 95%CI: 0.13;0.35  0.19, 95%CI: 0.11;0.31  0.30, 95%CI: 0.21;0.41  0.24, 95%CI: 0.16;0.34  0.19, 95%CI: 0.12;0.28  0.36, 95%CI: 0.23;0.52  0.34, 95%CI: 0.21;0.49  0.15, 95%CI: 0.07;0.29 |
| Preferred exercise frequency | *Top three exercise frequency*  *Germany*  Twice a week  Once a week  Three times a week  *The Netherlands*  Twice a week  Once a week  Three times a week/ I don’t know  *Poland*  Twice a week  Three times a week  Once every now and then  *Spain*  Twice a week  Three times a week  More than three times a week  *Sweden*  Twice a week  Three times a week  More than three times a week | 0.41, 95%CI: 0.31;0.52  0.27, 95%CI: 0.19;0.37  0.12, 95%CI: 0.07;0.21  0.46, 95%CI: 0.37;0.56  0.19, 95%CI: 0.12;0.28  0.09, 95%CI:0.05;0.17  0.32, 95%CI: 0.21;0.45  0.25, 95%CI: 0.16;0.38  0.13, 95%CI: 0.06;0.24  0.35, 95%CI: 0.26;0.46  0.22, 95%CI: 0.15;0.33  0.19, 95%CI: 0.12;0.28  0.40, 95%CI: 0.27;0.56  0.32, 95%CI: 0.20;0.47  0.23, 95%CI: 0.13;0.38 |
| Preferred exercise location | *Top three exercise location*  *Germany*  Public gym or community sports facility  No preference  Outdoors/ Physiotherapy practice  *The Netherlands*  Physiotherapy practice  Public gym or community sports facility  Outdoors  *Poland*  At home/ No preference  Physiotherapy practice  Public gym or community sports facility  *Spain*  Outdoors  Public gym or community sports facility  No preference  *Sweden*  Public gym or community sports facility  Physiotherapy practice  No preference | 0.34, 95%CI: 0.25;0.45  0.19, 95%CI: 0.12;0.29  0.15, 95%CI: 0.09;0.24  0.26, 95%CI: 0.18;0.36  0.25, 95%CI: 0.18;0.35  0.11, 95%CI: 0.06;0.19  0.21, 95%CI: 0.12;0.34  0.19, 95%CI: 0.10;0.32  0.18, 95%CI: 0.09;0.30  0.25, 95%CI: 0.17;0.35  0.17, 95%CI: 0.10;0.26  0.15, 95%CI: 0.09;0.24  0.36, 95%CI: 0.23;0.52  0.26, 95%CI: 0.14;0.41  0.15, 95%CI: 0.07;0.29 |
| Preferred exercise supervision | *Top three exercise supervision*  *Germany*  Fitness instructor or exercise professional  Physiotherapist  No preference  *The Netherlands*  Physiotherapist  No preference  Fitness instructor or exercise professional  *Poland*  Physiotherapist  Fitness instructor or exercise professional  No one  *Spain*  Physiotherapist  I don’t know  Fitness instructor or exercise professional  *Sweden*  Fitness instructor or exercise professional  Physiotherapist  No one | 0.42, 95%CI: 0.32;0.52  0.35, 95%CI: 0.26;0.46  0.10, 95%CI: 0.05;0.19  0.42, 95%CI: 0.33,0.52  0.16, 95%CI: 0.10;0.25  0.15, 95%CI: 0.09;0.24  0.52, 95%CI: 0.40;0.65  0.17, 95%CI: 0.09;0.30  0.14, 95%CI: 0.07;0.26  0.29, 95%CI: 0.21;0.39  0.18, 95%CI: 0.11;0.27  0.16, 95%CI: 0.09;0.25  0.40, 95%CI: 0.27;0.56  0.30, 95%CI: 0.18;0.45  0.15, 95%CI: 0.07;0.29 |
| Preferred exercise company | *Top three exercise company*  *Germany*  Cancer patients  No preference  No one  *The Netherlands*  No preference  General public  No one  *Poland*  No preference/ General public  No one  Cancer patients  *Spain*  No preference  Friends  Cancer patients  *Sweden*  No one  Cancer patients  General public | 0.26, 95%CI: 0.18;0.36  0.18, 95%CI: 0.11;0.28  0.16, 95%CI: 0.10;0.26  0.23, 95%CI: 0.15;0.32  0.21, 95%CI: 0.14;0.30  0.19, 95%CI: 0.12;0.28  0.21, 95%CI: 0.12;0.33  0.17, 95%CI: 0.09;0.30  0.16, 95%CI: 0.08;0.28  0.24, 95%CI: 0.16;0.34  0.18, 95%CI: 0.11;0.27  0.16, 95%CI: 0.09;0.25  0.23, 95%CI: 0.13;0.38  0.21, 95%CI: 0.11;0.36  0.15, 95%CI: 0.07;0.29 |

Table presents the proportion of participants endorsing a particular response. Proportions were calculated from the number of responses per country.
